# ERβ regulation of NF-κB activation in prostate cancer is mediated by HIF-1

**DOI:** 10.18632/oncotarget.5377

**Published:** 2015-10-02

**Authors:** Paul Mak, Jiarong Li, Sanjoy Samanta, Arthur M. Mercurio

**Affiliations:** ^1^ Department of Molecular, Cell and Cancer Biology, University of Massachusetts Medical School, Worcester, MA 01605, USA

**Keywords:** estrogen receptor beta, HIF-1, NFkB, prostate

## Abstract

We examined the regulation of NF-κB in prostate cancer by estrogen receptor β (ERβ) based on the inverse correlation between p65 and ERβ expression that exists in prostate carcinomas and reports that ERβ can inhibit NF-κB activation, although the mechanism is not known. We demonstrate that ERβ functions as a gate-keeper for NF-κB p65 signaling by repressing its expression and nuclear translocation. ERβ regulation of NF-κB signaling is mediated by HIF-1. Loss of ERβ or hypoxia stabilizes HIF-1α, which we found to be a direct driver of IKKβ transcription through a hypoxia response element present in the promoter of the IKKβ gene. The increase of IKKβ expression in ERβ-ablated cells correlates with an increase in phospho-IκBα and concomitant p65 nuclear translocation. An inverse correlation between the expression of ERβ and IKKβ/p65 was also observed in the prostates of ERβ knockout (BERKO) mice, Gleason grade 5 prostate tumors and analysis of prostate cancer databases. These findings provide a novel mechanism for how ERβ prevents NF-κB activation and raise the exciting possibility that loss of ERβ expression is linked to chronic inflammation in the prostate, which contributes to the development of high-grade prostate cancer.

## INTRODUCTION

Chronic inflammatory diseases are known to cause epithelial malignancies including prostate cancer [1, 2]. Recently, the Prostate Cancer Prevention Trial (PCPT) indicated that chronic intraprostatic inflammation influences the development of high-grade prostate cancer [3]. Therefore, understanding the mechanisms that contribute to chronic inflammation should facilitate the development of therapeutic approaches aimed at reducing the lethality of prostate cancer. Early studies revealed that ERβ signaling reduced systemic inflammation in some animal models [4, 5]. More recently, it was reported that ERβ-selective agonists can inactivate microglia and invading T cells by down regulating the expression of NF-κB [6]. Although cross-talk between ERβ and NF-κB is evident [7], the mechanism by which ERβ regulates the NF-κB pathway has not been resolved. In the resting stage of the canonical NF-kB pathway, the heterodimeric complex of RelA (p65) and p50 is sequestered in the cytoplasm in association with IκBα and is inactive. Once activated by external stimuli such as cytokines, lipopolysaccharide or viruses, IκB kinases (IKKβ) phosphorylate IκBα, which is degraded by the proteasome [8]. This cascade event allows the translocation of the p65.p50 complex to the nucleus where it regulates the transcription of its target genes. The key question is how does ERβ impact this process of NF-κB activation?

Work from our laboratory has established an important mechanistic link between ERβ and HIF-1. Specifically, we demonstrated that ERβ promotes the proteasomal degradation of HIF-1α by sustaining the expression of prolyl hydroxylase 2 (PHD2) [9, 10]. Consequently, loss of ERβ or function results in HIF-1α stabilization and HIF-1-mediated transcription. There is a functional link between the expression of HIF-1α and p65 in several cancers [11, 12], which is consistent with the fact that hypoxia and inflammation are common features of all solid tumors [13]. For these reasons, we examined the hypothesis that ERβ repression of the NF-kB pathway involves HIF-1α. Our data reveal that ERβ functions as a repressor of HIF-1α-mediated NF-kB activation and support the possibility that ERβ may contribute to the prevention of chronic inflammation in the prostate and prostate cancer.

## RESULTS

### Loss of ERβ promotes p65 expression and NF-κB activation

**Figure 1:**
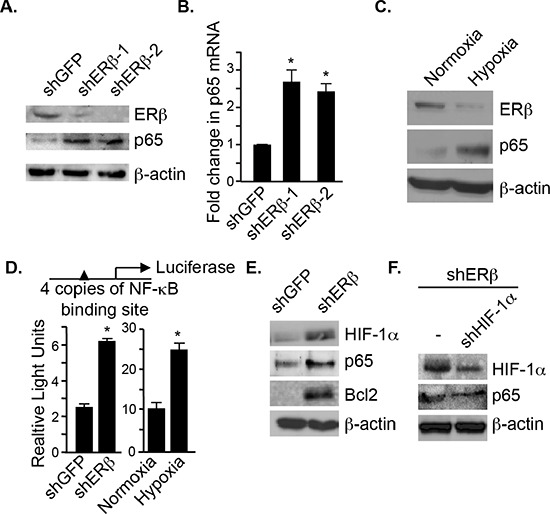
ERβ represses p65 expression and NF-κB activation Comparison of p65 protein **A.** and mRNA expression **B.** in control (shGFP) and ERβ-depleted PNT1a cells (shERβ-1 and shERβ-2). **C.** PNT1a cells were maintained in either normoxia or hypoxia for 20–24 hours and the expression of ERβ, p65 and actin was assessed by immunoblotting. **D.** An NF-κB luciferase reporter construct was used to compare NF-κB transcriptional activity between control and ERβ-depleted PNT1a cells, and PNT1a cells maintained in either normoxia or hypoxia for 20–24 hours. Data represent the average of three separate experiments with SEM indicated. (*p value: < 0.05). **E.** The expression of HIF-1α, p65 and Bcl2 was assessed in control and ERβ-depleted PNT1a cells by immunoblotting. **F.** An shRNA that targets HIF-1α was expressed in ERβ-depleted PNT1a cells and the expression of HIF-1α, p65 and actin was assessed by immunoblotting.

Loss of ERβ promotes an EMT in PNT1a cells, which are immortalized prostate epithelial cells [9]. Interestingly, we observed that ERβ-ablated PNT1a cells express high levels of p65 (both protein and mRNA) compared to control cells (shGFP) (Figure 1A and 1B). To substantiate the link between ERβ and p65, we exposed these cells to hypoxia, which diminishes ERβ expression [10]. As shown in Figure 1C, p65 expression is increased in hypoxic conditions with a concomitant decrease in ERβ. We next assessed NF-κB activation using an NF-κB luciferase reporter construct, which contains 4 copies of an NF-kB binding site. There was a greater than 2 fold increase in luciferase activity in both ERβ-ablated and hypoxic cells compared to their respective controls (shGFP and normoxic cells) (Figure 1D). These data indicate that ERβ regulates p65 expression and its activation. To understand the mechanism by which ERβ regulates p65 expression, we focused on HIF-1 because it can induce p65 expression [14]. HIF-1α is stabilized in ERβ-ablated PNT1a cells [9] resulting in an increase in p65 expression and its target gene, Bcl-2 (Figure 1E). More importantly, knocking-down HIF-1α in ERβ-ablated cells decreased p65 expression (Figure 1F).

### Loss of ERβ promotes p65 nuclear translocation

**Figure 2:**
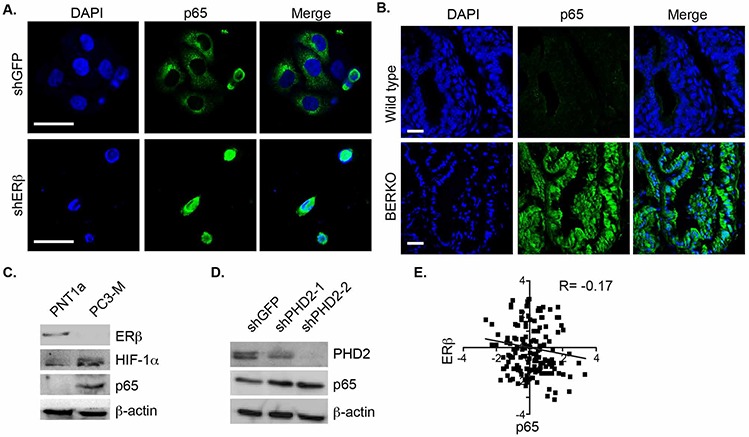
Analysis of ERβ and p65 localization and expression in prostate cells and tissues **A.** Control and ERβ-depleted PNT1a cells and **B.** Ventral prostates from 10 month old wild-type and BERKO mice were stained with a p65 antibody and DAPI to visualize nuclei, and analyzed by immunofluorescence microscopy. Scale bar: 50 μm. **C.** The expression of ERβ, HIF-1α and p65 was compared in PNT1a and PC3-M cells by immunoblotting. **D.** The expression of p65 was compared in control (shGFP) and PHD2-ablated PNT1a cells (shPHD2#1 and shPHD2#2) by immunoblotting. **E.** An inverse correlation between ERβ and p65 in a cohort of 82 prostate tumors was determined from analysis of the cBioportal database.

Given that the nuclear localization of p65 is associated with high Gleason grade prostate cancer [15, 16], we examined the relationship between p65 localization and ERβ expression in ERβ-ablated PNT1a cells. Immunofluorescence staining indicated that p65 is localized exclusively in the cytoplasm of control cells (shGFP) and in the nuclei of ERβ-ablated cells (shERβ) (Figure 2A). The notion that loss of ERβ promotes p65 nuclear translocation is supported by the analysis of the prostates from ERβ knockout (BERKO) mice. This analysis revealed nuclear p65 localization in BERKO prostates that was not evident in the age-matched, wild-type prostates (Figure 2B). Furthermore, an inverse correlation between the expression of ERβ and HIF-1α and p65 was detected by comparing normal prostate epithelial cells and PC3-M cells, which are an aggressive variant of PC3 cells [17] (Figure 2C). We also observed that diminishing PHD2 expression in PNT1a cells, which mimics the effect of ERβ loss and stabilizes HIF-1α [9], increased p65 expression (Figure 2D). A negative correlation between ERβ and p65 expression was also evident exists in a cohort of 87 human prostate tumors based on analysis of the cBioportal database (Figure 2E).

### ERβ regulates p65 nuclear translocation via the IKKβ/IκBα canonical pathway

**Figure 3:**
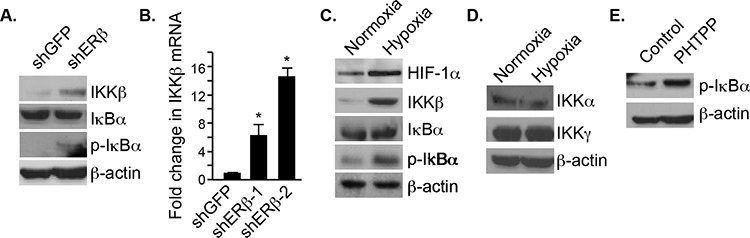
Regulation of IKKβ and pIκBα expression by ERβ and hypoxia **A.** The expression IKKβ, IκBα and pIκBα was compared in control and ERβ-depleted PNT1a cells by immunoblotting. **B.** The expression IKKβ mRNA was quantified in control and ERβ-depleted PNT1a cells by qPCR. **C.** PNT1a cells were maintained in either normoxia or hypoxia for 20–24 hours and the expression of HIF-1α, IKKb, IκBα pIκBα and actin was assessed by immunoblotting. **D.** PNT1a cells were maintained in either normoxia or hypoxia for 20–24 hours and the expression of IKKα and IKKγ was assessed by immunoblotting. **E.** The expression of pIκBα was assessed in PNT1a cells that had been treated with vehicle alone (control) or the ERβ antagonist PHTPP for 18 hours.

To understand how p65 nuclear translocation is regulated by ERβ signaling, we focused on the IKKβ/IκBα canonical pathway. Interestingly, an increase in IKKβ and *p*-IκBα expression was observed in ERβ-ablated PNT1a cells compared to control cells (Figure 3A). However, there was no difference in total IκBα expression. IKKβ mRNA levels were also elevated in ERβ-ablated cells compared to the control cells suggesting transcriptional regulation (Figure 3B). Hypoxia also increased the expression of both IKKβ and *p*-IκBα but not total IκBα (Figure 3C). The induction of IKKβ expression by hypoxia is specific because there was no change in the expression of IKKα and IKKγ (Figure 3D). Moreover, an increase in *p*-IκBα was observed when the cells were treated with a specific ERβ antagonist, PHTPP, supporting the possibility that the IKKβ/IκBα canonical pathway is regulated by ERβ (Figure 3E).

### IKKβ transcription is regulated by HIF-1α

**Figure 4:**
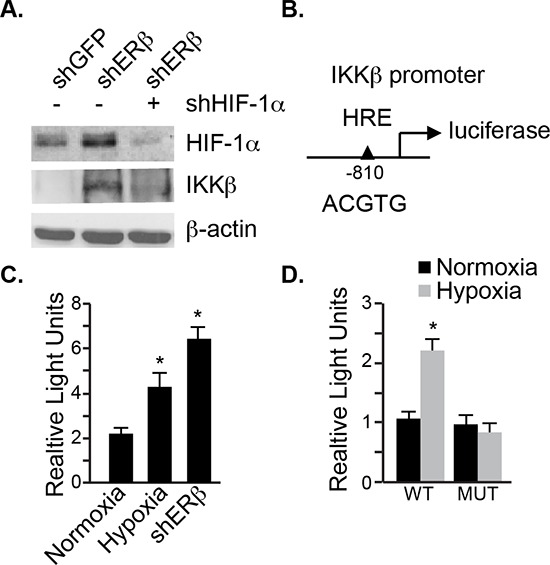
HIF-1α regulates IKKβ transcription **A.** The expression of HIF-1α and IKKβ was compared in control (shGFP), ERβ-depleted (shERβ) or ERβ-depleted PNT1a cells expressing a HIF-1α shRNA (shERβ/shHIF-1α) by immunoblotting. **B.** Schematic depicting a luciferase reporter construct containing a HIF-1 binding site in the IKKβ promoter. **C.** The reporter construct shown in **B** was used to compare IKKβ transcriptional activity between control and ERβ-depleted PNT1a cells. **D.** The oligonucleotide containing the hypoxia response element (HRE) as demonstrated in **C** was mutated from ACGTG to AAATG. Wild-type (wt) and HRE mutant (mut) reporter constructs were used to compare IKKβ transcriptional activity between control and ERβ-depleted PNT1a cells. Data represent three separate experiments with SEM indicated. (*p value: < 0.05).

To demonstrate that the increase of IKKβ expression in ERβ-ablated cells is mediated by HIF-1α, we knocked down HIF-1α and observed a dramatic reduction in IKKβ compared to the control cells (Figure 4A). This observation prompted us to examine whether the promoter of IKKβ contains a HIF-1 binding site. We found such a binding site (ACGTG) located at −810 bp upstream of the transcription start site (Figure 4B). Therefore, we cloned the promoter region from −1000 bp to +20 bp in a luciferase reporter plasmid, which was used to transfect ERβ-ablated cells or hypoxic cells. A 2–3 fold induction of normalized luciferase activity was seen in these cells compared to their respective controls (Figure 4C) providing evidence that this site functions as a hypoxia response element (HRE). Moreover, mutating this HRE to AAATG (HREm) in the reporter gene abrogated the hypoxia-mediated luciferase induction (Figure 4D). These data indicate that the expression of IKKβ is driven by HIF-1α in ERβ ablated cells.

### IKKβ expression is associated with high-grade prostate tumors

**Figure 5:**
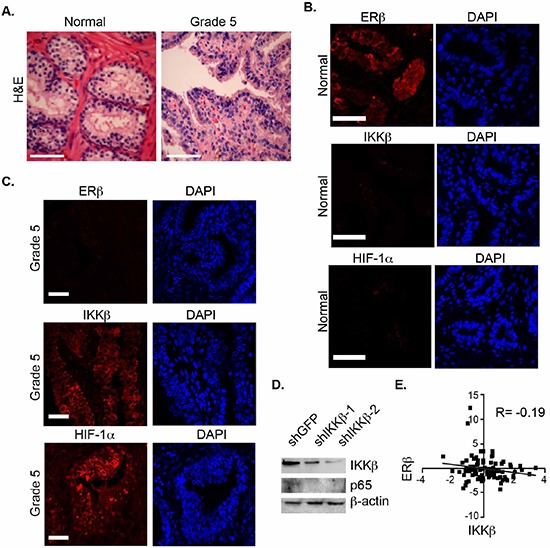
Analysis of ERβ, HIF-1α and IKKβ expression in prostate tissues **A.** H&E staining of normal prostate and primary Gleason grade 5 prostate carcinomas. **B, C.** These tissues were stained with antibodies specific for ERβ and IKKβ, counter-stained with DAPI to visualize nuclei, and analyzed by immunofluorescence microscopy. Scale bar: 50 μm. Six different specimens were examined with similar results. **D.** The expression of p65 was compared in control (shGFP) and IKKβ-ablated PC3-M cells (shIKKβ#1 and shIKKβ#2) by immunoblotting. **E.** An inverse correlation between the expression of ERβ and IKKβ was detected in a cohort of 65 prostate tumors was determined from analysis of the cBioportal database.

To assess whether an inverse correlation between ERβ and HIF-1α/IKKβ expression exists in prostate cancer, we compared their expression in Gleason grade 5 tumors and normal prostate epithelia by immunofluorescence staining. The H&E staining of these tissues is provided in Figure 5A. ERβ is expressed in normal epithelia but not in grade 5 tumors (Figure 5B) supporting our previous findings [10]. In marked contrast, high expression of IKKβ was observed in grade 5 tumors compared to normal epithelia (Figure 5C). A link between IKKβ and NF-κB activation in this context is indicated by our finding that knock-down of IKKβ expression in PC3-M cells, which exhibit properties of high-grade carcinoma, diminished p65 expression (Figure 5D). A negative correlation between ERβ and IKKβ expression was also evident in a cohort of 70 human prostate tumors based on analysis of the cBioportal database (Figure 5E).

## DISCUSSION

**Figure 6:**
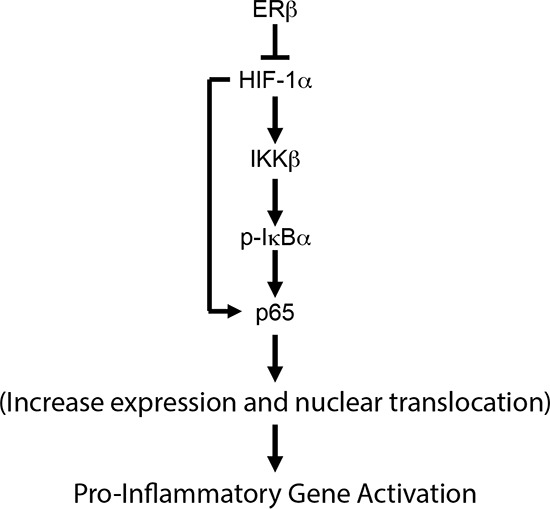
Schematic summarizing the major conclusion of the study We propose that ERβ functions as a gate-keeper of NF-κB activation by preventing the HIF-1-mediated activation of the IKKβ/pIκBα axis.

The results presented in this study provide a mechanism for how loss of ERβ, which occurs in high-grade prostate cancer, contributes to NF-κB activation. The core of this mechanism is that the loss of ERβ, which stabilizes HIF-1α [9], results in the HIF-1α mediated transcription of IKKβ and consequent nuclear translocation of p65 (Figure 6). These findings have important implications for development of chronic inflammation in prostate cancer and they highlight an unexpected anti-inflammatory role for the ability of ERβ to promote the degradation of HIF-1α.

Although many previous studies have described the ability of ERβ to inhibit NF-κB activation and reduce inflammation [4, 5], the specific mechanisms involved are less clear. Our functional characterization of a hypoxia responsive element (HRE) in the promoter of the IKKβ gene and demonstration that HIF-1α drives IKKβ transcription directly via this HRE provides such a mechanism. This mechanism is intimately associated with ERβ because it de-stabilizes HIF-1α [9, 10] and, consequently, prevents NF-kB activation. Interestingly, loss of ERβ had no effect on IKKα expression even though both IKKα and IKKβ phosphorylate IκBα [8]. A worthwhile question to pursue based on our findings is whether the ability of ERα to suppress NF-κB activation, which occurs in breast cancer [18], involves a similar mechanism. A contribution of ERα to NF-kB regulation is unlikely in the prostate, however, because ERα is not expressed in prostate epithelial or carcinoma cells.

The mechanism of NF-kB regulation by ERβ that we describe is likely to be one component of a more complex set of interactions. Specifically, there is evidence that IKKβ and NF-kB can promote HIF-1α transcription [11, 12]. This finding implies that a positive feedback loop involving HIF-1 and NF-kB exists, and that this feedback loop is under the repressive control of ERβ. Indeed, given the potential impact that this feedback loop can have on cells, the ability of ERβ to inhibit it becomes a critical regulatory event for preventing the consequences of HIF-1- and NF-kB-mediated transcription. There is also evidence that that a variant of ERβ, ERβ2, which lacks the ability to bind ligand and activate canonical ERβ gene expression, can interact with and stabilize HIF-1α in normoxic environments [19]. This finding is relevant to our work because ERβ2 is expressed in aggressive prostate cancers and it may contribute to the HIF-1/NF-kB positive feedback loop.

Our findings provide a possible mechanism for the increased NF-κB activation that is associated with high Gleason grade prostate tumors because these tumors also exhibit loss of ERβ and induction of HIF-1α expression [10]. This connection is very significant in light of the reports that chronic inflammation is associated with high-grade prostate cancer [20]. The link between loss of ERβ and chronic inflammation may also be relevant for more differentiated prostate tumors. Recently, we demonstrated that prostate tumors with a primary Gleason score of 3 are heterogeneous for ERβ expression. [21]. An interesting question going forward is whether grade 3 tumors that exhibit loss of ERβ are more inflammatory than those tumors that express ERβ. In pursuit of the mechanism involved, we discovered that tumors that were deficient in ERβ expression also exhibited loss of PTEN and that PTEN loss had a causal role in repressing ERβ [21]. It is worth noting in this context that PTEN loss promotes NF-κB activation in pancreatic ductal carcinoma [22] and loss of ERβ could be a critical factor in this activation.

## MATERIALS AND METHODS

### Cells and reagents

PNT1a cells were obtained from M. Littmann (Baylor College of Medicine, Houston). The human prostate cancer cell line, LNCaP was obtained from American Type Culture Collection (ATCC). PC3-M cells were obtained from R. C. Bergan (Northwestern University, Chicago). 3β-androstane-diol (3β-Adiol) and PHTPP experiments were performed by incubating cells with 3β-Adiol (5 μM; Sigma) or PHTPP (10 μM; Tocris) for 2–3 days. The generation of ERβ-ablated PNT1a cells, PHD2-ablated PNT1a cells and HIF-1α ablated cells has been described previously [9]. IKKβ ablated PC3-M cells were generated using shRNAs (Open Biosystems; TRCN0000018918 and TRCN0000018919). Stable cell transfectants were generated by puromycin or hygromycin selection (0.5 μg/mL for PNT1a and 2 μg/mL for PC3-M cells). The resultant ERβ, HIF-1α-ablated cells were used for subsequent experiments. For experiments involving hypoxia, cells were incubated with 100 μM cobalt chloride for 22–24 hours.

### Biochemical analyses

For immunoblotting, the following Abs were used p65, IKKα, IKKβ, IKKγ (Santa Cruz), HIF-1α (Novus) and β-actin (Sigma). Immune complexes were detected using enhanced chemiluminescence (ECL; Pierce). For quantitative real-time RT-PCR (qPCR), total RNA was extracted from cells using the TRI reagent (Sigma) and was reverse transcribed using reverse transcription reagents (Applied Biosystems) and analyzed by SYBR Green Master (Roche) using a real-time PCR system (ABI; Prism 7900HT Sequence Detection system). The expression of target genes was normalized to 18s RNA and analyzed by the comparative cycle threshold method (ΔΔCt). For luciferase assays, PC3-M cells were transfected with the desired plasmids and a Renilla luciferase construct to normalize for transfection efficiency. Luciferase assays were performed using Dual Glo luciferase assay system (Promega). Relative luciferase activity was calculated as the ratio of firefly luciferase to Renilla luciferase activity.

### Transgenic mice

ERβ knockout (BERKO) mice were generated by the Korach laboratory [23] and were purchased from The Jackson Laboratory. The knockout allele was maintained on a C57BL/6 background. The mice were used in these studies were 10 months old. Sections from these prostates and age-matched controls were processed for immunostaining as described below.

### Immunofluorescence staining

Murine prostate specimens from transgenic mice (see above) and human prostate cancer specimens, which were obtained from the Tissue Bank at the University of Massachusetts Medical School, were fixed in paraformaldehyde (4%), embedded in paraffin, sectioned (5 μM) and used for hematoxylin and eosin (H&E) and immunofluorescence staining. After antigen unmasking, the specimens were incubated in 10% serum in PBS for 30 minutes, washed for 3 min in PBST, and incubated with rabbit polyclonal p65 antibody (Santa Cruz), rabbit polyclonal IKKβ antibody (Santa Cruz) or HIF-1α monoclonal antibody (Novum) overnight at 4°C. The slides were washed 5 min with PBST and incubated 45 minutes in a dark chamber with the fluorochrome-conjugated secondary antibody (goat anti-rabbit conjugated Alexa Fluor 488, Life Sciences A-11008). Slides were washed and counterstained in the dark with DAPI (Invitrogen) for 10 minutes, washed with three changes of PBST and mounted under coverslips with aqueous mounting medium (Thermo Electron corp. Pittsburgh, PA). Results were analyzed using an LSM 710 Meta confocal microscope (Carl Zeiss MicroImaging Gmbh, Munich, Germany).

### Statistical analysis

Data are presented as the mean from three separate experiments ± SD. The Student *t* test was used to determine the significance of independent experiments. The criterion *P* < 0.05 was used to determine statistical significance.
